# Supplementary material for: Not only a territorial matter: The electoral surge of VOX and the anti-libertarian reaction
Source: PLoS One. 2023 Apr 7;18(4):e0283852. doi: 10.1371/journal.pone.0283852 (PMC10081776; doi:10.1371/journal.pone.0283852)
Supplement: S1 File — (DOCX) [file pone.0283852.s001.docx]

S1.- Methodological Appendix

**Table A1. Data Sheet of the PACIS post-electoral survey PACIS (2019)**

| Universe | Persons of 18 years or more who are residents of Andalusia |
| --- | --- |
| Sample framework | PACIS citizen panel |
| n | 1,037 interviews |
| Data collection | Sample selected from people belonging to PACIS. Contact made by email, SMS, and telephone. Questionnaire online (website) or telephone |
| Response rate | For the final sample of 1,037 interviews, 2,293 people from PACIS were selected (RR = 45.2%) |
| Sampling procedure | Stratified selection by age groups and sex of the PACIS members, the strata being proportional to the Andalusian population over 18 years of age |
| Consideration | Calibration by ranking method with the variables of sex and age on one hand, educational level on the other and size of the municipal population on the other, taking Andalusian population totals as a reference |
| Average interview time | 17 minutes |
| Maximum sample error | +/- 3% |
| Dates of field work | 1-28 March, 2019 |

**Table A2. Sample and population distribution**

|  |  | PACIS | | Official Statistics |
| --- | --- | --- | --- | --- |
|  |  | **Unweighted** | **Weighted** |  |
|  |  | **%** | **%** | **%** |
| Sex |  |  |  |  |
|  | Male | 49.2 | 48.8 | 48.8 |
|  | Female | 50.8 | 51.2 | 51.2 |
| Age |  |  |  |  |
|  | 18 to 29 y.o. | 16.1 | 16.5 | 16.5 |
|  | 30 to 44 y.o. | 27.8 | 28.0 | 28.0 |
|  | 45 to 59 y.o. | 29.0 | 27.8 | 27.8 |
|  | 60 and more | 27.1 | 27.6 | 27.6 |
| Education level* | |  |  |  |
|  | Primary | 11.6 | 25.2 | 25.2 |
|  | Secondary and vocational training | 53.3 | 57.2 | 57.2 |
|  | Higher education | 35.1 | 17.6 | 17.6 |
| Population size | |  |  |  |
|  | Less than 5,000 inhabitants | 7.1 | 13.6 | 13.6 |
|  | 5,000 to 10,000 inhabitants | 9.1 | 9.5 | 9.5 |
|  | 10,000 to 20,000 inhabitants | 12.1 | 16.4 | 16.4 |
|  | 20,000 to 100,000 inhabitants | 30.1 | 28.8 | 28.8 |
|  | More than 100,000 inhabitants | 41.8 | 31.7 | 31.7 |
| Labour status* | |  |  |  |
|  | Employed | 48.7 | 44.8 | 44.8 |
|  | Unemployed | 15.8 | 17.7 | 13.2 |
|  | Inactive | 35.1 | 37.5 | 42.0 |
|  | **n** | **1,037** | **1,037** | **6,270,353** |

Note: All data extracted from the Population Continuous Register (2018) except those marked with a star (*), that come from the Labour Force Survey (2018). Both datasets are conducted by the Spanish National Statistics Institute (INE).

**Table A3. Distribution of vote recall in the 2018 Andalusian elections for PACIS and CIS Post-election surveys and comparison with 2018 Andalusian election results**

|  | PACIS | | CIS | Election results | Differences | |
| --- | --- | --- | --- | --- | --- | --- |
|  | **Unweighted** | **Weighted** |  |  | **PACIS Weighted** | **CIS** |
|  | % | % | % | % | % | % |
| PSOE | 20.7 | 24.3 | 32.7 | 27.3 | -3.0 | +5.4 |
| PP | 17.5 | 16.3 | 17.4 | 20.3 | -4.0 | -2.9 |
| Ciudadanos | 22.7 | 21.0 | 16.4 | 17.9 | +3.1 | -1.5 |
| Adelante Andalucía | 22.0 | 20.6 | 16.4 | 15.8 | +4.8 | +0.6 |
| VOX | 8.4 | 8.2 | 7.1 | 10.7 | -2.5 | -3.6 |
| PACMA | 2.5 | 2.4 | 2.4 | 1.9 | +0.5 | +0.5 |
| Blanks | 2.5 | 3.3 | 4.7 | 1.5 | +1.8 | +3.2 |
| Nulls | 1.8 | 1.5 | 2.2 | 2.2 | -0.7 | +0.0 |
| Others | 2.0 | 2.3 | 0.8 | 2.3 | 0.0 | -1.5 |
| Total | 715 | 688 | 1,725 |  |  |  |
|  | **Mean Absolute Error** | | | | **2.3** | **2.1** |

**Table A4. Distribution of vote recall in the 2016 General election (vote in Andalusia) for PACIS and CIS Post-election surveys and comparison with 2016 General election results**

|  | PACIS | | CIS | Election results | Differences | |
| --- | --- | --- | --- | --- | --- | --- |
|  | **Unweighted** | **Weighted** |  |  | **PACIS Weighted** | **CIS** |
|  | % | % | % | % | % | % |
| PSOE | 28.0 | 32.5 | 42.2 | 30.9 | +1.6 | +11.3 |
| PP | 21.5 | 21.3 | 23.7 | 33.2 | -11.9 | -9.5 |
| Ciudadanos | 20.3 | 18.0 | 12.7 | 13.4 | +4.6 | -0.7 |
| Unidos Podemos | 23.5 | 20.9 | 16.1 | 18.4 | +2.5 | -2.3 |
| VOX | 0.5 | 0.4 | 0.2 | 0.2 | +0.2 | +0.0 |
| PACMA | 1.3 | 1.7 | 1.4 | 1.2 | +0.5 | +0.2 |
| Blanks | 2.2 | 2.5 | 2.1 | 0.9 | +1.6 | +1.2 |
| Nulls | 0.5 | 0.5 | 1.3 | 1.1 | -0.6 | +0.2 |
| Others | 2.1 | 2.2 | 0.4 | 0.7 | +1.5 | -0.3 |
| Total | **817** | **795** | **1,952** |  |  |  |
|  | **Mean Absolute Error** | | | | **2.8** | **2.9** |

**Table A5. List of all covariates used in Regression analyses**

| Hypothesis | Included in Table 2 models (T2) / Included in Table 3 models (T3) | Variable | Survey code | Recodification |
| --- | --- | --- | --- | --- |
| *Feminism* | T2 and T3 | Feminist ideas are fair | P25 | (1) “Disagree” and “Strongly disagree”  (0) “Agree” and “Strongly agree” |
|  | Only T2 | Feminism creates injustice for men | P26 | (1) “Agree” and “Strongly agree”  (0) “Disagree” and “Strongly disagree” |
| *Territorial* | T2 and T3 | Nationalist sentiment | P18 | (1) “Feels only Spanish” and “Feels more Spanish than Andalusian”  (0) “Feels as Spanish as Andalusian”, “Feels more Andalusian than Spanish” and “Feels only Andalusian” |
|  | T2 and T3 | State organisation: Centralisation-Autonomy | P19 | (1) “A State with a single government and no Autonomous Communities” and “A State in which the Autonomous Communities have less autonomy than at present”  (0) “A State with Autonomous Communities as at present”, “A State in which the Autonomous Communities have greater autonomy”, “A State that would recognise the possibility for Autonomous Communities to become independent states” |
|  | T2 and T3 | What to do about Catalonia | P20 | (1) “Be more heavy-handed in defending the unity of Spain”  (0) “Defending legality with as little repression as possible”,  “Combine the defence of legality with gestures of dialogue”,  “Negotiate until some political agreement is reached”,  “Allow Catalans to hold a referendum”, and  “I don't know what the solution is, but I'm fed up with it” |
| *Immigration* | T2 and T3 | Main problem of Andalusia | P1A and P1B | (1) Mentions immigration as the first or second problem  (0) Does not mention immigration as a problem |
|  | T2 and T3 | Building of a mosque in your neighbourhood | P23 | (1) “Disagree” and “Strongly disagree”  (0) “Agree” and “Strongly agree” |
|  | Only T2 | Assessment of immigration laws | P24 | “Too tolerant” (1) – “Too harsh” (5) (scale) |
| *Authoritarianism* | T2 and T3 | Our society does not need tougher government or stricter laws | P27 | (1) “Disagree” and “Strongly disagree”  (0) “Agree” and “Strongly agree” |
|  | Only T2 | Need for greater toughness on criminals | P28 | (1) “Strongly agree”  (0) “Agree”, “Disagree” and “Strongly disagree” |
|  | T2 and T3 | Priority of rights and freedoms in the fight against crime | P30 | “Prioritise the fight against crime” (0) – “Prioritise rights and freedoms” (10) (scale) |
| *Populism* | T2 and T3 | To solve Andalusian problems, people like me would do better than politicians | P2 | (1) “Agree” and “Strongly agree”  (0) “Disagree” and “Strongly disagree” |
|  | Only T2 | Politicians do not care what we think | P3 | (1) “Agree” and “Strongly agree”  (0) “Disagree” and “Strongly disagree” |
| *Control* | Only T3 | National Government rating (PSOE) | P5 | (1) “Bad” and “Very bad”  (0) “Neither good nor bad”, “Good” and “Very good” |
|  | Only T3 | Andalusia Government rating | P6 | (1) “Bad” and “Very bad”  (0) “Neither good nor bad”, “Good” and “Very good” |
|  | Only T3 | Religious affiliation and practice | PRELIG | *Reference category:* Atheists and non-believers  *Dummy variables:* Other beliefs; Non-practising Catholics or once a year; Catholics practising monthly or more |
|  | Only T3 | Sex | SEXO | Male (1), Female (0) |
|  | Only T3 | Ideological self-placement | IDEO | “Extreme left” (0) – “Extreme right” (10) (scale) |
|  | Only T3 | Age | EDAD | 18 – 99 (scale) |
|  | Only T3 | Educational level | NEST_R | *Reference category:* Illiterate, without studies and primary level studies  *Dummy variables:* Secondary or technical studies; University studies |
|  | Only T3 | Evaluation of the economic situation of your household over the last 12 months | NSOC | (1) “Average”, “Above average” and “Rich”  (0) “Below average”, “Almost poor” and “Poor” |
|  | Only Suppl. materials | Income | INGHG | (1) “No income at all”, (2) “300 Euros or less”, (3) “301-600”, (4) “601-900”, (5) “901-1,200”, (6) “1,201-1,800”, (7) “1,801-2,400”, (8) “2,401-3,000”, (9) “3,001-4,500”, (10) “4,501-6,000”, (11) “More than 6,000” |

Note 1: This table indicates which variables have been included in the initial models (Table 2), in the joint regression models (Table 3) or in both of them.

Note 2: Here we indicate the original categories that have been considered within each recoded category of the variables included. The DK and DA have been considered missing values in all variables.

**Table A6. Descriptive statistics of all** **covariates**

| Variable | | N | Mean | Std. Dev. | Min. | Max. |
| --- | --- | --- | --- | --- | --- | --- |
| *Feminist ideas are fair* | | 1,010 | 0.301 | 0.459 | 0 | 1 |
| *Feminism creates injustice for men* | | 1,002 | 0.416 | 0.493 | 0 | 1 |
| *Nationalist sentiment* | | 1,021 | 0.096 | 0.294 | 0 | 1 |
| *State organisation: Centralisation-Autonomy* | | 971 | 0.391 | 0.488 | 0 | 1 |
| *What to do about Catalonia* | | 1,006 | 0.247 | 0.432 | 0 | 1 |
| *Main problem of Andalusia* | | 1,036 | 0.133 | 0.340 | 0 | 1 |
| *Building of a mosque in your neighbourhood* | | 991 | 0.515 | 0.500 | 0 | 1 |
| *Assessment of immigration laws* | | 1,006 | 2.06 | 1.073 | 1 | 5 |
| *Our society does not need tougher government or stricter laws* | | 1,008 | 0.526 | 0.500 | 0 | 1 |
| *Need for greater toughness on criminals* | | 1,021 | 0.476 | 0.500 | 0 | 1 |
| *Priority of rights and freedoms in the fight against crime* | | 1,017 | 5.75 | 3.102 | 0 | 10 |
| *To solve Andalusian problems, people like me would do better than politicians* | | 1,021 | 0.661 | 0.474 | 0 | 1 |
| *Politicians do not care what we think* | | 1,029 | 0.865 | 0.342 | 0 | 1 |
| *National Government rating (PSOE)* | | 1,010 | 0.426 | 0.495 | 0 | 1 |
| *Andalusia Government rating* | | 1,021 | 0.518 | 0.500 | 0 | 1 |
| *Religious affiliation and practice*  *(Atheists and non-believers Cat. Ref.)* | *Other beliefs* | 1,014 | 0.048 | 0.214 | 0 | 1 |
|  | *Non-practising Catholics or once a year* | 1,014 | 0.449 | 0.498 | 0 | 1 |
|  | *Catholics practising monthly or more* | 1,014 | 0.195 | 0.396 | 0 | 1 |
| *Sex* | | 1,037 | 0.488 | 0.500 | 0 | 1 |
| *Ideological self-placement* | | 1,001 | 4.74 | 1.958 | 0 | 10 |
| *Age* | | 1,037 | 48.37 | 16.410 | 19 | 89 |
| *Educational level*  *(Illiterate, without studies and primary level studies Cat. Ref.)* | *Secondary or technical studies;* | 1,037 | 0.573 | 0.495 | 0 | 1 |
|  | *University studies* | 1,037 | 0.176 | 0.381 | 0 | 1 |
| *Evaluation of the economic situation of your household over the last 12 months* | | 1,027 | 0.585 | 0.493 | 0 | 1 |
| *Income* | | 996 | 5.50 | 1.939 | 1 | 11 |

**Table A7. Correlation heat map of all covariates**

| P25 | 1 |  |  |  |  |  |  |  |  |  |  |  |  |  |  |  |  |  |  |  |  |  |  |  |  |
| --- | --- | --- | --- | --- | --- | --- | --- | --- | --- | --- | --- | --- | --- | --- | --- | --- | --- | --- | --- | --- | --- | --- | --- | --- | --- |
| P26 | 0.335 | 1 |  |  |  |  |  |  |  |  |  |  |  |  |  |  |  |  |  |  |  |  |  |  |  |
| P18 | 0.123 | 0.086 | 1 |  |  |  |  |  |  |  |  |  |  |  |  |  |  |  |  |  |  |  |  |  |  |
| P19 | 0.171 | 0.208 | 0.198 | 1 |  |  |  |  |  |  |  |  |  |  |  |  |  |  |  |  |  |  |  |  |  |
| P20 | 0.21 | 0.214 | 0.094 | 0.271 | 1 |  |  |  |  |  |  |  |  |  |  |  |  |  |  |  |  |  |  |  |  |
| P1A/P1B | 0.089 | 0.108 | 0.032 | 0.073 | 0.14 | 1 |  |  |  |  |  |  |  |  |  |  |  |  |  |  |  |  |  |  |  |
| P23 | 0.176 | 0.174 | 0.036 | 0.206 | 0.221 | 0.137 | 1 |  |  |  |  |  |  |  |  |  |  |  |  |  |  |  |  |  |  |
| P24 | -0.291 | -0.349 | -0.072 | -0.288 | -0.351 | -0.218 | -0.457 | 1 |  |  |  |  |  |  |  |  |  |  |  |  |  |  |  |  |  |
| P27 | 0.212 | 0.079 | 0 | 0.122 | 0.231 | 0.058 | 0.21 | -0.274 | 1 |  |  |  |  |  |  |  |  |  |  |  |  |  |  |  |  |
| P28 | 0.15 | 0.187 | 0.061 | 0.165 | 0.227 | 0.064 | 0.15 | -0.326 | 0.238 | 1 |  |  |  |  |  |  |  |  |  |  |  |  |  |  |  |
| P30 | -0.089 | -0.155 | -0.05 | -0.199 | -0.16 | -0.02 | -0.172 | 0.242 | -0.172 | -0.253 | 1 |  |  |  |  |  |  |  |  |  |  |  |  |  |  |
| P2 | 0.049 | 0.065 | -0.015 | 0.054 | 0.077 | 0.011 | 0.054 | -0.101 | 0.094 | 0.083 | -0.012 | 1 |  |  |  |  |  |  |  |  |  |  |  |  |  |
| P3 | 0.049 | 0.07 | -0.052 | 0.063 | 0.09 | -0.018 | 0.09 | -0.107 | 0.052 | 0.083 | -0.008 | 0.331 | 1 |  |  |  |  |  |  |  |  |  |  |  |  |
| P5 | 0.308 | 0.227 | 0.026 | 0.224 | 0.235 | 0.004 | 0.145 | -0.289 | 0.177 | 0.139 | -0.093 | 0.058 | 0.056 | 1 |  |  |  |  |  |  |  |  |  |  |  |
| P6 | 0.197 | 0.13 | 0.017 | 0.165 | 0.142 | 0.013 | 0.128 | -0.145 | 0.191 | 0.138 | -0.187 | 0.145 | 0.129 | 0.48 | 1 |  |  |  |  |  |  |  |  |  |  |
| PRELIG | 0.029 | 0.018 | -0.015 | -0.068 | -0.019 | 0.015 | 0.005 | 0.004 | -0.009 | -0.014 | 0.004 | 0.052 | 0.063 | -0.036 | 0.007 | 1 |  |  |  |  |  |  |  |  |  |
| PRELIG | 0.109 | 0.098 | 0.014 | 0.099 | 0.09 | 0.042 | 0.124 | -0.196 | 0.096 | 0.127 | -0.113 | 0.021 | 0.006 | -0.006 | 0.032 | -0.204 | 1 |  |  |  |  |  |  |  |  |
| PRELIG | 0.042 | 0.002 | 0.037 | 0.087 | 0.109 | 0.112 | 0.082 | -0.107 | 0.036 | -0.002 | 0.011 | -0.005 | -0.052 | 0.069 | -0.018 | -0.112 | -0.442 | 1 |  |  |  |  |  |  |  |
| SEXO | -0.049 | 0.045 | 0.053 | 0.119 | -0.038 | -0.072 | -0.012 | 0.049 | -0.107 | 0.025 | -0.052 | -0.072 | 0.001 | -0.005 | 0.046 | -0.045 | -0.047 | -0.137 | 1 |  |  |  |  |  |  |
| IDEO | 0.262 | 0.325 | 0.117 | 0.302 | 0.316 | 0.124 | 0.211 | -0.31 | 0.202 | 0.214 | -0.096 | 0.022 | 0.016 | 0.323 | 0.134 | -0.01 | 0.141 | 0.211 | -0.069 | 1 |  |  |  |  |  |
| EDAD | -0.046 | -0.073 | 0.07 | 0.094 | -0.003 | 0.016 | 0.081 | -0.016 | -0.098 | -0.098 | 0.152 | -0.067 | -0.106 | -0.108 | -0.157 | -0.077 | 0.015 | 0.279 | -0.113 | -0.003 | 1 |  |  |  |  |
| NEST_R | 0.046 | 0.092 | -0.06 | -0.067 | 0.094 | 0.013 | 0.054 | -0.07 | 0.05 | 0.125 | -0.073 | -0.04 | 0.018 | 0.069 | 0.047 | 0.027 | -0.021 | -0.024 | -0.012 | 0.053 | -0.142 | 1 |  |  |  |
| NEST_R | -0.032 | -0.064 | 0.02 | 0.01 | -0.044 | -0.055 | -0.1 | 0.141 | 0.025 | -0.046 | -0.006 | 0 | 0.01 | 0.025 | 0.085 | -0.01 | -0.041 | -0.046 | 0.085 | -0.049 | -0.301 | -0.539 | 1 |  |  |
| NSOC | -0.044 | -0.026 | 0.068 | 0.018 | 0.03 | -0.001 | -0.008 | 0.022 | 0.03 | -0.036 | -0.036 | -0.019 | -0.014 | -0.028 | -0.016 | -0.118 | -0.034 | 0.107 | 0.015 | 0.127 | 0.005 | -0.052 | 0.171 | 1 |  |
| INGHG | 0.016 | 0.002 | 0.038 | 0.075 | 0.007 | -0.046 | -0.025 | 0.03 | -0.019 | 0.03 | -0.013 | -0.097 | 0.009 | 0.069 | 0.065 | -0.065 | -0.087 | 0.03 | 0.187 | 0.064 | -0.037 | 0.006 | 0.263 | 0.423 | 1 |
|  | P25 | P26 | P18 | P19 | P20 | P1A /P1B | P23 | P24 | P27 | P28 | P30 | P2 | P3 | P5 | P6 | PRELIG | PRELIG | PRELIG | SEXO | IDEO | EDAD | NEST_R | NEST_R | NSOC | INGHG |

**Table A8. Linear Regression Model for the Probability of voting for VOX (extended data information)**

|  | | B | Beta coefficient | TOL | VIF |
| --- | --- | --- | --- | --- | --- |
| *Feminist ideas are unfair* | | 0.920***  (0.188) | 0.141 | 0.812 | 1.232 |
| *Nationalist sentiment* | | 0.214  (0.266) | 0.022 | 0.915 | 1.092 |
| *Recentralisation of the State* | | 0.811***  (0.185) | 0.131 | 0.749 | 1.335 |
| *Heavy-handed approach to defending unity in Spain* | | 0.632**  (0.199) | 0.092 | 0.807 | 1.239 |
| *Immigration as an Andalusian problem* | | 0.705**  (0.237) | 0.079 | 0.941 | 1.063 |
| *Rejection of the building of a mosque in your neighbourhood* | | 0.423*  (0.170) | 0.070 | 0.847 | 1.181 |
| *Need for a tougher government* | | 0.607***  (0.171) | 0.101 | 0.841 | 1.189 |
| *Fight against crime vs. rights and freedoms* | | - 0.092**  (0.027) | - 0.094 | 0.872 | 1.147 |
| *People like me would do better than politicians* | | 0.034  (0.168) | 0.005 | 0.943 | 1.060 |
| *National Government rating (PSOE)* | | 0.278  (0.195) | 0.046 | 0.650 | 1.537 |
| *Andalusia Government rating* | | 0.375*  (0.185) | 0.062 | 0.716 | 1.397 |
| *Religion (Atheists and Non-believers Cat. Ref.)* | *Other beliefs* | 0.658  (0.401) | 0.045 | 0.898 | 1,114 |
|  | *Non-practising Catholics or once a year* | - 0.080  (0.195) | - 0.013 | 0.649 | 1,542 |
|  | *Catholics practising monthly or more* | 0.083  (0.262) | 0.010 | 0.617 | 1,621 |
| *Sex* | | 0.446**  (0.163) | 0.074 | 0.920 | 1.087 |
| *Ideological self-placement* | | 0.544***  (0.048) | 0.343 | 0.718 | 1.392 |
| *Age* | | - 0.006  (0.006) | - 0.033 | 0.719 | 1.390 |
| *Educational level (Illiterate, Without studies and Primary Studies Cat. Ref.)* | *Secondary or technical studies* | - 0.325  (0.215) | - 0.053 | 0.546 | 1,833 |
|  | *University studies* | - 0.140  (0.268) | - 0.018 | 0.550 | 1,817 |
| *Evaluation of the economic situation in your household* | | 0.303^+^  (0.167) | 0.049 | 0.913 | 1.095 |
| Constant | | - 1.792** |  |  |  |
| n | | 864 |  |  |  |
| R^2^ adjusted | | 0.421 |  |  |  |

***p < 0.001; **p < 0.01; *p < 0.05; +p < 0.1. In parentheses the standard errors. The ANOVA test is significant at the level of 0.001.

**Table A9. Linear Regression Model for the Probability of voting for VOX (all variables included)**

|  | | B | Beta coefficient | TOL | VIF |
| --- | --- | --- | --- | --- | --- |
| *Feminist ideas are unfair* | | 0.877***  (0.197) | 0.134 | 0.748 | 1.336 |
| *Feminism creates injustice for men* | | 0.321^+^  (0.184) | 0.052 | 0.754 | 1.326 |
| *Nationalist sentiment* | | 0.161  (0.268) | 0.017 | 0.901 | 1.109 |
| *Recentralisation of the State* | | 0.789***  (0.190) | 0.127 | 0.729 | 1.372 |
| *Heavy-handed approach to defending unity in Spain* | | 0.613**  (0.204) | 0.089 | 0.781 | 1.280 |
| *Immigration as an Andalusian problem* | | 0.693**  (0.240) | 0.078 | 0.922 | 1.084 |
| *Rejection of the building of a mosque in your neighbourhood* | | 0.391*  (0.182) | 0.065 | 0.753 | 1.328 |
| *Assessment of immigration laws* | | 0.040  (0.097) | 0.014 | 0.572 | 1.748 |
| *Need for a tougher government* | | 0.618***  (0.175) | 0.102 | 0.810 | 1.235 |
| *Need for greater toughness on criminals* | | 0.012  (0.177) | 0.002 | 0.798 | 1.254 |
| *Fight against crime vs. rights and freedoms* | | - 0.096**  (0.028) | - 0.097 | 0.838 | 1.193 |
| *People like me would do better than politicians* | | 0.109  (0.179) | 0.017 | 0.848 | 1.180 |
| *Politicians do not care what we think* | | - 0.136  (0.259) | - 0.015 | 0.830 | 1.205 |
| *National Government rating (PSOE)* | | 0.214  (0.200) | 0.035 | 0.635 | 1.576 |
| *Andalusia Government rating* | | 0.422**  (0.188) | 0.070 | 0.708 | 1.412 |
| *Religion (Atheists and Non-believers Cat. Ref.)* | *Other beliefs* | 0.621  (0.402) | 0.043 | 0.887 | 1.128 |
|  | *Non-practising Catholics or once a year* | - 0.085  (0.200) | - 0.014 | 0.626 | 1.598 |
|  | *Catholics practising monthly or more* | 0.024  (0.267) | 0.003 | 0.609 | 1.642 |
| *Sex* | | 0.383**  (0.168) | 0.063 | 0.883 | 1.132 |
| *Ideological self-placement* | | 0.555***  (0.050) | 0.349 | 0.695 | 1.438 |
| *Age* | | - 0.005  (0.006) | - 0.026 | 0.709 | 1.411 |
| *Educational level (Illiterate, Without studies and Primary Studies Cat. Ref.)* | *Secondary or technical studies* | - 0.309  (0.221) | - 0.050 | 0.527 | 1.898 |
|  | *University studies* | - 0.124  (0.281) | - 0.016 | 0.506 | 1.974 |
| *Evaluation of the economic situation in your household* | | 0.256  (0.183) | 0.042 | 0.778 | 1.285 |
| *Income* | | 0.005  (0.048) | 0.003 | 0.725 | 1.379 |
| Constant | | - 1.932**  (0.639) |  |  |  |
| n | | 841 |  |  |  |
| R^2^ adjusted | | 0.427 |  |  |  |

***p < 0.001; **p < 0.01; *p < 0.05; +p < 0.1. In parentheses the standard errors. The ANOVA test is significant at the level of 0.001.

**Table A10. Logistic Regression Model for Vote recall for VOX (extended data information)**

|  | |  |  | **95% C.I. for Exp (B)** | |
| --- | --- | --- | --- | --- | --- |
|  | | **B** | **Exp (B)** | **Lower** | **Upper** |
| *Feminist ideas are unfair* | | 1.219**  (0.392) | 3.385 | 1.570 | 7.299 |
| *Nationalist sentiment* | | 0.171  (0.482) | 1.186 | 0.462 | 3.049 |
| *Recentralisation of the State* | | 1.117*  (0.449) | 3.056 | 1.269 | 7.363 |
| *Heavy-handed approach to defending unity in Spain* | | - 0.654  (0.421) | 0.520 | 0.228 | 1.186 |
| *Immigration as an Andalusian problem* | | 1.467**  (0.430) | 4.338 | 1.868 | 10.073 |
| *Rejection of the building of a mosque in your neighbourhood* | | 0.390  (0.426) | 1.478 | 0.642 | 3.403 |
| *Need for a tougher government* | | 0.581  (0.444) | 1.787 | 0.749 | 4.265 |
| *Fight against crime vs. rights and freedoms* | | - 0.067  (0.065) | 0.935 | 0.823 | 1.062 |
| *People like me would do better than politicians* | | - 0.100  (0.393) | 0.905 | 0.419 | 1.955 |
| *National Government rating (PSOE)* | | 1.173*  (0.507) | 3.232 | 1.196 | 8.730 |
| *Andalusia Government rating* | | 0.857^+^  (0.503) | 2.356 | 0.879 | 6.311 |
| *Religion (Atheists and Non-believers Cat. Ref.)* | *Other beliefs* | 1.769*  (0.834) | 5.863 | 1.143 | 30.067 |
|  | *Non-practising Catholics or once a year* | - 0.893^+^  (0.517) | 0.410 | 0.149 | 1.128 |
|  | *Catholics practising monthly or more* | - 0.664  (0.584) | 0.515 | 0.164 | 1.616 |
| *Sex* | | 0.658^+^  (0.386) | 1.932 | 0.906 | 4.119 |
| *Ideological self-placement* | | 0.463***  (0.119) | 1.589 | 1.259 | 2.005 |
| *Age* | | - 0.021  (0.015) | 0.979 | 0.951 | 1.008 |
| *Educational level (Illiterate, Without studies and Primary Studies Cat. Ref.)* | *Secondary or technical studies* | - 1.070^+^  (0.568) | 0.343 | 0.113 | 1.044 |
|  | *University studies* | - 1.286^+^  (0.665) | 0.276 | 0.075 | 1.017 |
| *Evaluation of the economic situation in your household* | | 0.424  (0.422) | 1.528 | 0.668 | 3.491 |
| ***Constant*** | | - 6.017*** | 0.002 |  |  |
| ***n*** | | 610 |  |  |  |
| ***R^2^*** | | 0.458 |  |  |  |

***p < 0.001; **p < 0.01; *p < 0.05; +p < 0.1. In parentheses the standard errors. The Hosmer and Lemeshow test is not significant (> 0.05). Global percentage of classification: 91.3%.

**Table A11. Logistic Regression Model for Vote recall for VOX (all variables included)**

|  | |  |  | **95% C.I. for Exp (B)** | |
| --- | --- | --- | --- | --- | --- |
|  | | **B** | **Exp (B)** | **Lower** | **Upper** |
| *Feminist ideas are unfair* | | 0.808^+^  (0.432) | 2.245 | 0.962 | 5.236 |
| *Feminism creates injustice for men* | | 1.882**  (0.608) | 6.565 | 1.994 | 21.612 |
| *Nationalist sentiment* | | 0.029  (0.513) | 1.029 | 0.377 | 2.814 |
| *Recentralisation of the State* | | 1.211*  (0.479) | 3.358 | 1.313 | 8.588 |
| *Heavy-handed approach to defending unity in Spain* | | - 0.717  (0.448) | 0.488 | 0.203 | 1.175 |
| *Immigration as an Andalusian problem* | | 1.072*  (0.459) | 2.921 | 1.188 | 7.182 |
| *Rejection of the building of a mosque in your neighbourhood* | | 0.343  (0.454) | 1.409 | 0.578 | 3.429 |
| *Assessment of immigration laws* | | - 0.241  (0.290) | 0.786 | 0.445 | 1.387 |
| *Need for a tougher government* | | 0.592  (0.470) | 1.808 | 0.720 | 4.541 |
| *Need for greater toughness on criminals* | | - 0.266  (0.443) | 0.767 | 0.322 | 1.828 |
| *Fight against crime vs. rights and freedoms* | | - 0.056  (0.070) | 0.945 | 0.824 | 1.084 |
| *People like me would do better than politicians* | | 0.011  (0.438) | 1.011 | 0.428 | 2.387 |
| *Politicians do not care what we think* | | - 0.664  (0.643) | 0.515 | 0.146 | 1.817 |
| *National Government rating (PSOE)* | | 0.986^+^  (0.535) | 2.680 | 0.940 | 7.642 |
| *Andalusia Government rating* | | 0.902^+^  (0.529) | 2.465 | 0.874 | 6.949 |
| *Religion (Atheists and Non-believers Cat. Ref.)* | *Other beliefs* | 1.243  (0.891) | 3.466 | 0.604 | 19.885 |
|  | *Non-practising Catholics or once a year* | - 1.233*  (0.566) | 0.291 | 0.096 | 0.882 |
|  | *Catholics practising monthly or more* | - 0.523  (0.620) | 0.593 | 0.176 | 1.998 |
| *Sex* | | 0.733^+^  (0.429) | 2.082 | 0.897 | 4.832 |
| *Ideological self-placement* | | 0.440***  (0.126) | 1.552 | 1.213 | 1.987 |
| *Age* | | - 0.014  (0.015) | 0.987 | 0.957 | 1.017 |
| *Educational level (Illiterate, Without studies and Primary Studies Cat. Ref.)* | *Secondary or technical studies* | - 0.706  (0.616) | 0.494 | 0.148 | 1.652 |
|  | *University studies* | - 1.051  (0.725) | 0.350 | 0.084 | 1.449 |
| *Evaluation of the economic situation in your household* | | 0.474  (0.497) | 1.606 | 0.606 | 4.252 |
| *Income* | | - 0.139  (0.139) | 0.870 | 0.662 | 1.143 |
| ***Constant*** | | - 5.554** | 0.004 |  |  |
| ***n*** | | 601 |  |  |  |
| ***R^2^*** | | 0.512 |  |  |  |

***p < 0.001; **p < 0.01; *p < 0.05; +p < 0.1. In parentheses the standard errors. The Hosmer and Lemeshow test is not significant (> 0.05). Global percentage of classification: 92.4%.

**Table A12. Robustness check 1: Logistic Regression Model for Vote recall for VOX vs PP-Ciudadanos**

|  | |  |  | **95% C.I. for Exp (B)** | |
| --- | --- | --- | --- | --- | --- |
|  | | **B** | **Exp (B)** | **Lower** | **Upper** |
| *Feminist ideas are unfair* | | 1.120**  (0.403) | 3.063 | 1.392 | 6.744 |
| *Nationalist sentiment* | | - 0.040  (0.484) | 0.961 | 0.372 | 2.483 |
| *Recentralisation of the State* | | 0.822^+^  (0.464) | 2.276 | 0.917 | 5.647 |
| *Heavy-handed approach to defending unity in Spain* | | - 0.670  (0.423) | 0.512 | 0.223 | 1.172 |
| *Immigration as an Andalusian problem* | | 1.545**  (0.451) | 4.688 | 1.937 | 11.349 |
| *Rejection of the building of a mosque in your neighbourhood* | | 0.049  (0.449) | 1.051 | 0.436 | 2.532 |
| *Need for a tougher government* | | 0.413  (0.463) | 1.512 | 0.610 | 3.743 |
| *Fight against crime vs. rights and freedoms* | | - 0.059  (0.069) | 0.943 | 0.824 | 1.079 |
| *People like me would do better than politicians* | | 0.016  (0.404) | 1.016 | 0.460 | 2.242 |
| *National Government rating (PSOE)* | | 1.088*  (0.523) | 2.970 | 1.065 | 8.277 |
| *Andalusia Government rating* | | 0.608  (0.508) | 1.837 | 0.679 | 4.968 |
| *Religion (Atheists and Non-believers Cat. Ref.)* | *Other beliefs* | 2.233*  (0.985) | 9.325 | 1.353 | 64.248 |
|  | *Non-practising Catholics or once a year* | - 1.038^+^  (0.529) | 0.354 | 0.126 | 0.999 |
|  | *Catholics practising monthly or more* | - 0.735  (0.586) | 0.480 | 0.152 | 1.511 |
| *Sex* | | 0.737^+^  (0.398) | 2.090 | 0.959 | 4.556 |
| *Ideological self-placement* | | 0.366**  (0.127) | 1.442 | 1.124 | 1.850 |
| *Age* | | - 0.026^+^  (0.015) | 0.974 | 0.946 | 1.003 |
| *Educational level (Illiterate, Without studies and Primary Studies Cat. Ref.)* | *Secondary or technical studies* | - 1.182**  (0.594) | 0.307 | 0.096 | 0.983 |
|  | *University studies* | - 1.490**  (0.687) | 0.225 | 0.059 | 0.866 |
| *Evaluation of the economic situation in your household* | | 0.318  (0.429) | 1.374 | 0.593 | 3.183 |
| ***Constant*** | | - 3.851**  (1.450) |  |  |  |
| ***n*** | | 314 |  |  |  |
| ***R^2^*** | | 0.376 |  |  |  |

***p < 0.001; **p < 0.01; *p < 0.05; +p < 0.1. In parentheses the standard errors. The Hosmer and Lemeshow test is not significant (> 0.05). Global percentage of classification: 85.8%.

**Table A13. Robustness check 2: Logistic Regression Model for Vote recall for VOX vs PP**

|  | |  |  | **95% C.I. for Exp (B)** | |
| --- | --- | --- | --- | --- | --- |
|  | | **B** | **Exp (B)** | **Lower** | **Upper** |
| *Feminist ideas are unfair* | | 0.896^+^  (0.498) | 2.450 | 0.924 | 6.497 |
| *Nationalist sentiment* | | 0.308  (0.605) | 1.361 | 0.416 | 4.453 |
| *Recentralisation of the State* | | 0.411  (0.551) | 1.509 | 0.512 | 4.444 |
| *Heavy-handed approach to defending unity in Spain* | | - 0.801  (0.522) | 0.449 | 0.161 | 1.248 |
| *Immigration as an Andalusian problem* | | 1.524**  (0.550) | 4.591 | 1.563 | 13.489 |
| *Rejection of the building of a mosque in your neighbourhood* | | - 0.097  (0.554) | 0.907 | 0.306 | 2.686 |
| *Need for a tougher government* | | 0.938  (0.573) | 2.555 | 0.832 | 7.847 |
| *Fight against crime vs. rights and freedoms* | | - 0.051  (0.082) | 0.950 | 0.808 | 1.116 |
| *People like me would do better than politicians* | | - 0.013  (0.464) | 0.987 | 0.397 | 2.452 |
| *National Government rating (PSOE)* | | 1.430**  (0.665) | 4.180 | 1.134 | 15.402 |
| *Andalusia Government rating* | | 0.120  (0.632) | 1.127 | 0.326 | 3.894 |
| *Religion (Atheists and Non-believers Cat. Ref.)* | *Other beliefs* | 1.679  (1.392) | 5.362 | 0.350 | 82.137 |
|  | *Non-practising Catholics or once a year* | - 2.209**  (0.793) | 0.110 | 0.023 | 0.520 |
|  | *Catholics practising monthly or more* | - 2.230**  (0.833) | 0.108 | 0.021 | 0.550 |
| *Sex* | | 0.831^+^  (0.478) | 2.296 | 0.900 | 5.857 |
| *Ideological self-placement* | | 0.146  (0.155) | 1.157 | 0.855 | 1.567 |
| *Age* | | - 0.028  (0.018) | 0.972 | 0.939 | 1.007 |
| *Educational level (Illiterate, Without studies and Primary Studies Cat. Ref.)* | *Secondary or technical studies* | - 1.401*  (0.707) | 0.246 | 0.062 | 0.986 |
|  | *University studies* | - 1.668*  (0.820) | 0.189 | 0.038 | 0.942 |
| *Evaluation of the economic situation in your household* | | 0.608  (0.532) | 1.837 | 0.648 | 5.209 |
| ***Constant*** | | - 0.299  (1.762) | 0.742 |  |  |
| ***n*** | | 163 |  |  |  |
| ***R^2^*** | | 0.402 |  |  |  |

***p < 0.001; **p < 0.01; *p < 0.05; +p < 0.1. In parentheses the standard errors. The Hosmer and Lemeshow test is significant (> 0.05). Global percentage of classification: 78.2%.
