# Supplementary material for: Not only a territorial matter: The electoral surge of VOX and the anti-libertarian reaction
Source: PLoS One. 2023 Apr 7;18(4):e0283852. doi: 10.1371/journal.pone.0283852 (PMC10081776; doi:10.1371/journal.pone.0283852)
Supplement: Attachment — Submitted filename: Response to reviewers.docx [file pone.0283852.s002.docx]

Dear PLoS ONE Editor and Reviewers,

Thank you very much for taking the time to review our work and give us your valuable feedback. We have edited the manuscript to address your concerns and we feel that the outcome has improved as a result of this revision. We answer to each of your comments below and describe the relevant changes that we have made to the manuscript.

## Editorial Comment

1. Please ensure that your manuscript meets PLOS ONE's style requirements, including those for file naming.

## Answer and Revisions

We have completely revised our files to match PONE style and format guidelines.

## Editorial Comment

2. We note that the grant information you provided in the ‘Funding Information’ and ‘Financial Disclosure’ sections do not match.

When you resubmit, please ensure that you provide the correct grant numbers for the awards you received for your study in the ‘Funding Information’ section.

## Answer and Revisions

We have checked the grant information and corrected it when resubmitting the files.

## Editorial Comment

3. Please note that in order to use the direct billing option the corresponding author must be affiliated with the chosen institute. Please either amend your manuscript to change the affiliation or corresponding author, or email us at plosone@plos.org with a request to remove this option.

## Answer and Revisions

We have requested to remove this option.

## Editorial Comment

4. Please include captions for your Supporting Information files at the end of your manuscript, and update any in-text citations to match accordingly.

## Answer and Revisions

Captions for Supporting Information appendix are now included at the end of the main manuscript text. In-text citations now refer to specific supporting tables, where appropriate.

## Reviewer's Questions

1. Is the manuscript technically sound, and do the data support the conclusions?

Reviewer #1: Yes

Reviewer #2: Partly

## Response and Revisions

Following your suggestions, we have rechecked our statistical analyses and added the regression models including all our independent variables as supporting information (Tables A9 and A11 in S1 Methodological Appendix). We have also computed a correlation heatmap (Table A7 in S1 Methodological Appendix) and made transparent in the main text our model specification (lines 341 to 346, p. 17 in the unmarked manuscript). We have modified the description of table 2 in order to better explain the use of r2 adjusted to rank alternative hypotheses according to their explanatory power (lines 317 to 330, p.15) and clarified the links between results and conclusions throughout the main text. We feel that the paper has improved in transparency, robustness and clarity as a result of this process.

## Reviewer's Questions

3. Have the authors made all data underlying the findings in their manuscript fully available?

Reviewer #1: Yes

Reviewer #2: No

## Response and Revisions

Together with our initial submission, we uploaded an anonymized data set as Supporting Information that allowed replication of our analyses. However, since then we have also published the complete underlying dataset and documentation of this survey, that is now open-access and available for download here: (https://doi.org/10.20350/digitalCSIC/14804).

## Reviewer comments

## Reviewer #1

The authors report that co-linearity is not an issue for their study. However, I still wonder to what extent single variables of interest correlate with each other. This is mostly because I believe that some of the questions do not only tap into their foreseen theoretical concept, but potentially grasp parts of the variation due to other concepts. Most importantly, left-right self-placement and attitudes towards feminism, authoritarian attitudes and nationalism (especially the item “state organization” calling for harsher action against regional dissenters) and immigration and left-right self-placements might not be measuring completely different phenomena. It would be great if the authors could show a co-linearity plot in the appendix in addition to the VIF and TOL reported to give readers an intuitive inside into potential issues with co-linearity.

## Response and Revisions

We have added a correlation heatmap of all covariates as table A7 in S1 Methodological Appendix. Several variables that moderately correlate (0.3-0.4) with others indicators underlying the same hypothesis or dimension (P3 for populism, P24 for immigration, P26 for feminism, P28 for authoritarianism or household income) had already been excluded from our final models. Overall, discounting correlation between dummy variables computed from the same categorical variable (i.e. education level, NEST-R, or religious practice, PRELIG), the heatmap portraits a similar scenario as co-linearity measures (VIF and TOL) that we presented in the models. All of them show that co-linearity is not affecting our models in a significant manner. Even if ideology, as you pointed out, is somehow correlated to many of our attitudinal variables, the strength of this association is limited (the highest ones being around 0.3).

## Reviewer #1

I wonder, why are not all variables included in the final model that are listed in Table A5? The authors state in a note to Table A5 that they have not included all variables into the main models. Does it change the results if they do?

This might be especially interesting for the populism item. Is your finding robust to using the other item that populism does not seem to explain (much) of the variance in support for VOX? I would not be surprised, and the authors acknowledge that their test of populism is not a particularly rigorous one focusing on only one question. Taking the second item into account might be a stronger test.

## Response and Revisions

We have included the full models as Tables A9 and A11 in S1 Methodological Appendix. As you can see, including all variables do not alter our results significantly. Moreover, the loss of explanatory power in the final model as compared to the complete model is almost negligible for the linear regression (0.427 to 0.421), and limited for the logistic regression (0.512 to 0.458, global percentage of classification from 92.4% to 91.3%). We have also computed the models exchanging the items excluded in each dimension and this does not improve them or produce relevant changes in the results. We do not show those additional models in the appendix for the sake of simplicity.

## Reviewer #1

It would also be great if the authors could add a short codebook to the supplementary information so that reviewers (and others) can replicate their findings more easily. As of now, it is sometimes difficult to tell which variables link to which item in the study, even although I can read Spanish.

## Response and Revisions

The complete dataset of the survey, together with codebooks in Spanish and English, have been published in the open access institutional repository of the Spanish National Research Council, that follows best practices in data sharing and management. All the information regarding this survey dataset can be accessed here: <https://doi.org/10.20350/digitalCSIC/14804> . We have added the variable labels in the original dataset to table A5 in the Supplementary Information to ease replication of the analyses and findings discussed in the manuscript.

## Reviewer #1

There are some typos that the authors might want to take care of:

p.8 “Marcos-Marne et al 2021)suggests”

p.11 “gained parliamentary representation in Spain since [the] 1979 general election”

p.13 “within the margin of error of the survey (+-3%).Finally”

## Response and Revisions

Thank you very much for such a detailed and careful revision. We have corrected these typos in the new version.

## Reviewer #2

Whilst I find the paper, on the whole, interesting and methodologically straightforward, I am not entirely convinced that the paper currently makes enough of an independent contribution to merit publication. There is scope for a more concise and theoretically streamlined iteration of the paper to perhaps be published as a research note.

1. My primary concern is related to the paper’s claim to a contribution. The paper begins by signalling that there is more to the story than the Catalan crisis and that these factors need to be considered too. The paper then goes on, however, to highlight the vast catalogue of work that highlights the different factors that scholars have considered.

## Response and Revisions

Perceived novelty of research results is not a relevant publication criterion for PLoS journals. However, we do think that our paper indeed makes relevant and novel contributions to this area of research. Following your suggestion, we have clarified them in the manuscript (lines 65 to 79, pages 3 and 4) and justify further in the lines below.

We depart from an original dataset that is the outcome of a survey that we specifically designed to test if the main explanatory factors for the rise of other Western European radical right-wing parties applied to the election where Vox got represented in a parliament for the first time in Spain (the 2018 Andalusian regional election). Our results – using two alternative measures of support for VOX – show that the preferences and attitudes related to the territorial conflict do matter, but their relevance is clearly reduced when we add alternative explanations.

The main contribution of our work is twofold. First, our analysis provides empirical evidence on the importance that several factors, for which there are limited data in Spain, had to explain VOX performance in that particular election. These factors are authoritarian attitudes and opposition to feminism, with the latter showing an important explanatory power of support for VOX, even after controlling for other alternative factors. Second, by being able to model the different factors in the same dataset, we can rank them according to their relevance explaining the electoral surge of this party. This is something that can’t be done using secondary data, such as CIS post-election surveys or political barometers, because they lack variables that would contribute to build a more complete explanation of the vote for individual parties, particularly the most recent ones such as VOX.

Different factors explaining “who votes for Vox” have been considered in the literature using different sources of data regarding different elections, but most of them have based their analyses in secondary data that lacked information about some of these explanations (Turnbull-Dugarte 2019; Ortiz 2019: Turnbull-Dugarte et al. 2020). Other commercial surveys have tackled a wider array of factors, but their results have been reported in the daily press in a necessarily simple and descriptive way (El País, 2018). We have tried to be really careful by reviewing and giving credit to all those works in our manuscript.

Regarding anti-feminism, most previous research has approached this subject from the supply side. Some of this research hypothesises on the effects that the anti-feminism in the party manifesto and leaders’ discourse may have in the observed gender gap in support for this party (Rama et al. 2021: page 71; Turnbull-Dugarte, 2019; Mendes and Dennison, 2021). Other authors have described and analysed the anti-feminist discourse produced by this party, via quantitative and content analysis of the party’s manifestos (Cabeza et al. 2021; Fernández Suárez, 2021), parliamentary speeches (Fernández Suárez, 2021), publications in social media (Bernárdez- Rodal et al. 2020, Luque and Cano, 2020) or all of them (Álvarez Benavides and Jiménez Aguilar, 2021).

From the demand side, and under the same hypothesis used here of an anti-libertarian reaction, Rama et al. 2021, analysing European Social Survey data, show the link between support for Vox and opposition to LGBT+ rights, specifically opposing same-sex marriage.

As far as we are aware, our paper is the first academic analysis that empirically proves the link between having anti-feminist attitudes and voting for Vox. Moreover, our data allow to control for other alternative explanations and rank the importance of all these factors according to their explanatory power.

## Reviewer #2

The paper mentions the work by Marcos-Marne an colleagues published in Politics. This paper looks at the issue of gender and women’s issues, theorising and demonstrating it does indeed play a role. Given this extant literature, what is the paper’s central novel thesis and/or finding? If it is that views on the perceived “fairness” of feminism is distinct from views on women’s issues, then this may well be more clearly argued.

## Response and Revisions

The paper published by Marcos Marne et al. in Politics in 2021 does not deal with gender and women issues. The paper considers whether demand-side populism plays a role in VOX’s vote share. As they put it: They *“combine holistic grading of key speeches and electoral manifestos, with the analysis of innovative survey data to respond to two main research questions: are populist ideas central to the electoral discourse of VOX; and is VOX more electorally attractive to voters who themselves display stronger populist attitudes?”*.

In this paper, preferences that tap into lesbian, gay, bisexual, transgender (LGBT) and women’s rights are included as a control in the regression models together as a “new variable that represent the latent structure of preferences of the individuals on the topic” and are in fact statistically non-significant in both models, computed to explain the declared probability of voting for VOX and VOX voting intention (see model 5 column in tables 3 and 4 of the paper, pages 9 and 10). Thus, that paper makes a relevant contribution, but a quite different one from our text, which empirically proves the link between having anti-feminist attitudes and voting for Vox.

## Reviewer #2

2. I find the somewhat summative (and long) list of hypotheses not necessarily helpful for the paper. Is this a paper of “who votes for VOX?” – that is how the long list of hypotheses currently reads and as mentioned, that seems well answered. My recommendation would be focus on the main explanatory variable of the paper and make this more a independent variable focused paper that hones in on what the author’s identify as being important and make a strong case for why it should be considered. Of course, authors write papers and not reviewers, but I provide this recommendation in order to help the authors find a mechanism via which they can more clearly sell what the value-added component of their contribution is. Essentially the summative hypotheses are just controls for your main variables of interest.

## Response and Revisions

Yes, this is a paper that intends to answer who voted for Vox in the election where this party got represented in a parliament for the first time in Spain (the 2018 Andalusian regional election). Published articles on this issue (Turnbull-Dugarte, 2019 and Ortiz, 2019) employed data from CIS post-election survey, a high-quality source of data, that follows a standard questionnaire but did not include variables allowing to test factors that have proved important when explaining the emergence of radical right-wing parties in Europe. Our data, coming from a different high-quality post-election survey that was designed to explain *ad-hoc* the results of that election, allow us to show a more complete picture of the factors that played a role on that occasion and to rank them according to their explanatory power. When presenting our findings, we have underlined the factor that was most novel in this research area, anti-feminist attitudes, but treating the rest of factors as mere controls would imply, in our opinion, loosing valuable insights on the subject. We appreciate the recommendation, but consider that the current structure reflects better the goals and contributions of the paper.

## Reviewer #2

3. It would be useful for the authors to make more of a case for the external validity of the case they consider. Andalucian elections in 2018 feel very remote in time from this vantage point, we have had numerous national, supranational, regional, and municipal elections since then and VOX has had varying levels of success across these elections. Can the claims of what mattered in region X and time t-x wield external validity now? I don’t necessarily think that they don’t, but I do have some concerns about this.

## Response and Revisions

We answer to the question of generalizability and external validity in lines 523 to 536 (pages 24 and 25) of the manuscript:

*“First, regarding the generalizability of the results, the Andalusian elections of 2018 represented the electoral surge of VOX. As such, they represent a great opportunity to understand how their electorate was formed for the first time. Our analysis has shown that, from the very first minute, their success was based on quite more than territorial politics. Some of their defining characteristics at that time have probably changed today in Andalusia or Spain. However, the similarities shown through the text with the situation of many other European radical right parties, as well as VOX’s relative electoral stability since then (Rama et al 2021), suggests that there are not so many singularities in this 2018 electoral process, and that there are many anticipatory signs of what VOX continues to represent in Spain and in comparative perspective. For example, regarding our main hypothesis, a recent survey (El País, 2022) shows that two anti-feminist proposals (limiting abortion rights and abolish the Law against gender-based violence) are the second and third VOX proposals more easily identified by the Spanish population, thus showing that this idea continues to be a central part of the party’s identity”.*

## Reviewer #2

4. Finally, I have concerns of the substantive interpretation of the findings in relation to the broader claims of “why” VOX was successful. The paper effectively demonstrates that anti-feminist believes wield a sizeable and significant independent effect on support for the party. Is that equitable to the events of Catalonia. In a counterfactual scenario where the events of the Catalan crisis didn’t occur, is it the paper’s claim that anti-feminists’ positions would have been enough to explain VOX’s success? This is somewhat implicit throughout the paper.

## Response and Revisions

This is an interesting counterfactual question. The truth is that we don’t know if VOX mobilization of anti-feminist positions would have been enough to be present in the Andalusian parliament after the 2018 regional election. All that we can safely say looking at our data is that the emergence of VOX in Andalusia can be explained as a consequence of multiple factors where the variables associated with the territorial conflict were the most important, even after controlling for the main alternative explanations and that the reaction against the values of the “libertarian left”, particularly against feminism but also immigration, also played a relevant role. Our data allow us to explicitly rank the importance of the different factors contributing to explain the vote for Vox in the 2018 Andalusian election, which we do in the Discussion and conclusions section.

## Reviewer #2

1. The discussion of the different r2 between different models was a bit unusual and I’m not convinced the interpretation that accompanies this comparison is doing what the paper’s authors believe it is doing.

## Response and Revisions

We have modified the description of table 2 and rewritten that section in order to better explain the use of R^2^ adjusted to rank alternative hypotheses according to their explanatory power (lines 317 to 330, p.15).

Thanks again to all of you for what we feel has been a productive review process.
