# Supplementary material for: Not only a territorial matter: The electoral surge of VOX and the anti-libertarian reaction
Source: PLoS One. 2023 Apr 7;18(4):e0283852. doi: 10.1371/journal.pone.0283852 (PMC10081776; doi:10.1371/journal.pone.0283852)
Supplement: Attachment — Submitted filename: Response to Reviewers.docx [file pone.0283852.s003.docx]

Dear PLoS ONE Editor,

First of all, we would like to thank the reviewers for taking the time to review our work and give us their valuable feedback. We have edited the manuscript to address their concerns following the suggestions offered. As a result of this revision, we feel that the outcome has improved again. We answer to each of their comments below and describe the relevant changes that we have made to the manuscript.

## Editorial Comment

1. Please review your reference list to ensure that it is complete and correct. If you have cited papers that have been retracted, please include the rationale for doing so in the manuscript text or remove these references and replace them with relevant current references. Any changes to the reference list should be mentioned in the rebuttal letter that accompanies your revised manuscript. If you need to cite a retracted article, indicate the article’s retracted status in the References list and also include a citation and full reference for the retraction notice.

## Answer and Revisions

We have completely revised our Reference List. There are no papers that have been retracted among them nor have we made any additional changes to our Reference List.

## Reviewer's Questions

3. Has the statistical analysis been performed appropriately and rigorously?

Reviewer #1: No

Reviewer #2: Yes

## Response and Revisions

Following R1's suggestions, we have made our description and interpretation of results of Table 2 more precise. As the reviewer indicated, the adjusted R^2^ is better interpreted in terms of the goodness-of-fit of that model to the DV. Our interpretation of the data can now be read in that way (p.15, lines 318-334). We have also included in Table 2 the AIC (Akaike Information Criterion) and BIC (Bayesian Information Criterion) values for these models (p.15). Since these models are not nested nor do they contain the same number of variables, these measures are appropriate to determine the goodness-of-fit of each and to compare these different models. As can be seen, both values show us a similar ordering of the variables to that of adjusted R^2^ and allow us to rank them before introducing them into the joint model.

As for our interpretation of the Negelkerke R^2^, we have also avoided talking about the percentage of variance explained of the dependent variable (p.17, lines 347-348; p.18, lines 386-387). This information only allows us to assess the fit of the model, and this is how it can be read in the revised version of the paper.

## Reviewer's Questions

5. Is the manuscript presented in an intelligible fashion and written in standard English?

PLOS ONE does not copyedit accepted manuscripts, so the language in submitted articles must be clear, correct, and unambiguous. Any typographical or grammatical errors should be corrected at revision, so please note any specific errors here.

Reviewer #1: No

Reviewer #2: Yes

## Response and Revisions

In order to make the text easier to read and understand, we have clarified the point that had been identified as confusing by R1 (p.17, lines 351-354). In particular, the interpretation of the contribution of the different hypotheses in the discussion has been refined (p.21, lines 441-443; p.21, lines 454-457; p.22, lines 471-473). This new reading is made easier by the changes made to the description and interpretation of Table 2, as mentioned above. In this way, not only is the interpretation of the results obtained more precise, but it also helps the reader to properly follow the explanation given. In addition, some typos have been corrected, which R1 kindly pointed out to us for correction in the revised manuscript.

## Reviewer comments

## Reviewer #1

Minor things regarding wording

I find the sentence “all the covariates included in the remaining hypotheses…” (p. 17, line 355) confusing, because there has been no hypothesis discussed briefly before and I struggle to understand which hypotheses, if not all, the authors refer to exactly.

Some of the conclusions are not fully clear to me. In your discussion, you state that “the variables associated with the territorial conflict are those which contribute most to explaining the vote for VOX” (p. 21, line 445-6), but I do not see that reflected in Table 2 nor in the beta coefficients presented in Table A9. Maybe you could rephrase this sentence to reflect that, e.g. anti-feminist attitudes, seem to play an at least equally important role – which I assume was also one of the main arguments you wanted to make. As of know, it reads as a general statement that all else being equal, positions regarding the Spanish territorial conflicts are most important. Similarly, the discussion states that “a rejection of immigration has been the third most important factor” (p. 22, line 475), although this is not reflected in the beta coefficients in Table A9. More than two factors show a larger beta coefficient, e.g. the fight against crime.

## Response and Revisions

We agree with R1 that the sentence (s)he points out could be a bit confusing. For this reason, we have integrated the explanation together with the previous paragraph (p.17, lines 351-354). In this way, it does not lose the point of what is being discussed or lead to confusion by referring to the hypotheses.

Moreover, the interpretation of the specific role of each hypothesis in the discussion has benefited from the fact that we have been more precise with the language when describing and interpreting our results regarding the adjusted R^2^ in Table 2. In this sense, some sentences that Reviewer 1 pointed out in the discussion section have been modified so that they fully stick to the joint reading of the results of the two tables and its results (Table 2 and Table 3). Both tables show the relevance of the variables relating to the territorial hypothesis (H2.1) (particularly that relating to preferences regarding the territorial structure of the State) and those relating to the feminism hypothesis (H1). Both their fit to the model (Table 2) and their contribution to the explanation of DV (Table 3) indicate their importance. The variables relating to the immigration (H2.2) or authoritarianism hypothesis (H2.3) also show a good contribution to the explanation of the VOX vote. Both tables, moreover, do not support the populism hypothesis (H2.4) as relevant in this explanation. We have therefore been careful to explain the relevance and contribution of all these variables in explaining the VOX vote (p.21, lines 441-443; p.21, lines 454-457; p.22, lines 471-473).

## Reviewer #1

Typos

p. 2, line 37 “these voters [have not been] so [different] from voters of other European ...”

p. 17, line 342-3: “that moderately correlate with otherS indicators”

p. 17, line 345: “does not change [] the results [substantially]”? (English not my mother tongue, so maybe I am wrong here.)

p. 22, line 479: “feminism, [an] issue that VOX …”

p. 19, line 410: “the variable which focus[es]...”

In Table A9, A11 and A12 of the appendix, the authors use the “,” instead of the “.” for their decimals (e.g. 0,877 instead of 0.877).

## Response and Revisions

We have corrected each of these typos pointed out by R1, both in the text (p.2, line 37; p.17, line 343; p.17, line 345; p.19, line 406; p.22, line 466) and in the Supplementary Materials (Table A6, Table A7, Table A9, Table A10, Table A11, Table A12 and Table A13). We would like to thank her/him for providing us with such a detailed review. This feedback has proven invaluable in enhancing the initial version of the paper, and has helped us identify minor details that we had previously overlooked.

## Reviewer #1

Discussion of R² values

I would like to rise one issue that reviewer 2 had previously mentioned as a minor point. Your response and adjustments point out that you want to rank models in Table 2 based on their explanatory power using the adjusted R². From what I learned, the adjusted R², in contrast to the R², cannot be (easily) interpreted as the percentage of variance explained by the model because it does not only take variance into account, but also the number of variables included in the model. While thinking about possible solutions easy to implement during a second round of reviewing, other questions arose:

What are the underlying models for Table 2? Most importantly: which set of variables do they contain? Do they contain control variables or only variables for each hypothesis (feminism, territorial, …)? For each hypothesis, do the models contain the full set of items (Table A5) or only the item included in the regression analyses in Table 3?

Depending on the answers to these questions, the authors might consider 1) using the simple R² and compare the models based on that (if the models contain the same number of variables, and if the set of respondents would be the same in each model), 2) just describing the simple R² as percentage of variance explained but refrain from comparing models, or 3) tone down the language on the percentage of variance explained by the model. If you want to stick to the model comparison, I would recommend to use additional criteria such as AIC and BIC to compare the models with regard to their goodness of fit because the models are not nested nor do they seem to contain the same number of variables.

I can recommend A Guide to Modern Econometrics by Marno Verbeek for these things, which I regularly use myself, especially pages R², AIC, BIC and model comparisons.

I do not think that the model comparison and Table 2 is super important to answer your research question, which is why I would be ok with what-ever solution you find. I would just like to encourage you to be more precise in describing and interpreting the models presented in Table 2 or alternative models you might choose. It would also be great if the authors could add the information regarding the questions above to the supplementary material.

Similarly, I would like to ask the authors to reconsider their interpretation of the Negelkerke R². I have never used it before, but as far as I understood, this measure, again is not based on the variance (only) and thus, should not be interpreted as the percentage of the variance explained by the model. Again, one solution might be to simply delete the statement that this is equal to “explaining 46% of the variability of vote recall” (p. 19, line 389-90) and simply point out that the model fit seems great.

## Response and Revisions

We have reviewed in great detail the discussion surrounding the interpretation of R^2^ values as underlined in the first round of reviews by Reviewer 2 and now by Reviewer 1. Since, as both of them noted, adjusted R^2^ cannot easily be interpreted in terms of the percentage of explained variance of the DV, in the description and interpretation of Table 2 we have chosen to stick to the explanation that we can give: the goodness-of-fit of each model to the explanation of the dependent variable. Our models are not embedded, nor do they contain the same number of variables (see Table A5 in the Supplementary Materials), so we could not go for the first option suggested by R1. Since our intention was to stick to the model comparison, we opted for the third option (p.15, lines 318-334). As suggested, we have included the values of AIC and BIC in Table 2 (p.15) as additional criteria, in order to strengthen our interpretation and increase the transparency of the comparative analysis of our models.

We have clarified in the text (p.15, lines 321-322) and in the Supplementary Materials (Table A5) which variables compose the different models analysed in Table 2. Thus, the reader can better and more quickly understand the results of each model shown there.

To make all this clearer, we have also toned down throughout the text the references to the percentage of variance explained by the models. This concerns both the interpretation of the adjusted R^2^ (p.15, lines 318-334) and the Negelkerke R^2^ (p.17, lines 347-348; p.18, lines 386-387). The recommended book, "A Guide to Modern Econometrics" by Marno Verbeek, has been really helpful in enabling us to provide a more precise description and interpretation of the adjusted R^2^ values.

## Reviewer #2

The authors have made some updates to their initial submission, largely in response to some of the concerns raised by reviewer 1.

My initial primary concern with the paper was that the article did not make any original contribution to knowledge based on empirical findings or on theoretical arguments. I made some suggestions about changing the focus of the paper to make it more theoretically rich as opposed to empirically descriptive, but the authors have opted to retain their original structure.

As I mentioned in my earlier report, it is up to authors to write paper rather than reviewers, so I respect the author(s)’ decision here even if I do not, unfortunately, believe it makes their paper particularly citable.

I have no empirical qualms with the evidence presented which is straightforward and transparent.

## Response and Revisions

We would like to thank R2 for all the constructive feedback she/he has provided in both review rounds. The discussion regarding R^2^ has been a pivotal aspect of this round, one which we consider both enriching and gratifying. It has allowed us to expand our personal learning and as a result, the paper has been significantly improved.

Thanks again to all of you for what we feel has been a very productive review process.
